# Is a Hospital Pageant Practicable?

**Published:** 1921-05-21

**Authors:** W. M. Wilcox

**Affiliations:** Secretary. East London Hospital for Children, Shadwell, E. 1.


### IS A HOSPITAL PAGEANT PRACTICABLE?

*To the Editor of* The Hospital.

Sir,—Your proposal to hold a joint pageant for all the London hospitals appeals to me very strongly. I am sure the time has come when individual efforts must give way to the co-operative method. Mr. Inman's suggestion of a Hospital Week, supported by the three Funds and organised by a Committee representing all the London hospitals, is an excellent one, and, if worked with what our American friends call "pep," should be productive of great results. Let us have a meeting to talk it over as soon as possible.

Faithfully yours,

W. M. Wilcox, Secretary.

East London Hospital for Children, Shadwell, E. 1.

